# Glutamate administration is associated with aggravated atherosclerosis and altered expression of RAPGEF1, OPN, and MYL7 in High-Fat Diet-Fed ApoE⁻/⁻ Mice

**DOI:** 10.1371/journal.pone.0354719

**Published:** 2026-07-28

**Authors:** Xiuli Cheng, Fanshu Dai, Dilraba Mahmut, Xingya Huang, Qin Wang, Biao Zhang

**Affiliations:** 1 Department of Clinical Laboratory, Huanhu Hospital Affiliated to Tianjin Medical University, Tianjin, China; 2 Tianjin Key Laboratory of Cerebral Vascular and Neurodegenerative Diseases, Tianjin, China; Tokai University School of Medicine: Tokai Daigaku Igakubu Daigakuin Igaku Kenkyuka, JAPAN

## Abstract

**Objective:**

This study aims to examine whether oral glutamate administration is associated with accelerated atherosclerosis progression in high-fat diet-fed ApoE ⁻ / ⁻ mice and to identify candidate protein associated with this process.

**Methods:**

Atherosclerosis (AS) was induced in ApoE ⁻ / ⁻ mice by feeding a high-fat diet (HFD) for three months. The mice were randomly divided into four groups: (1) the AS-GLU group, receiving glutamate with HFD; (2) the AS group, receiving HFD with placebo; (3) the Glu group, receiving glutamate with normal chow; and (4) the Control group, receiving normal chow with a placebo. Atherosclerotic lesions were assessed using Lillie's oil red O staining and histological analysis. LC-MS/MS based proteomics profiling was employed to identify differentially expressed proteins, with subsequent validation by Western blotting and qPCR.

**Results:**

Glutamate administration alone (Glu group) induced minimal plaque formation. The combination of HFD and glutamate (AS-GLU) was associated with a greater atherosclerotic plaque area compared to HFD alone (AS group). Proteomics identified RAPGEF1, OPN, and MYL7 as proteins whose expression was increased in the AS-GLU group compared to AS group. These associations were confirmed by Western blot and qPCR.

**Conclusions:**

This study shows that oral glutamate administration, in the context of a high-fat diet in ApoE ⁻ / ⁻ mice, is associated with exacerbated atherosclerosis and increased expression of RAPGEF1, OPN, and MYL7. These proteins represent candidate molecules associated with glutamate exposure, but their causal role remains to be determined. Serum glutamate levels were not significantly elevated, and lipid profile changes may also contribute to the observed associations.

## 1. Introduction

Atherosclerosis (AS) is a chronic vascular inflammatory disease characterized by the accumulation of lipid deposits, immune cells infiltrates, and fibrous elements in the arterial wall, leading to the formation of atherosclerotic plaques [1]. As the primary etiological contributor to cardiovascular diseases (CVDs), which remain the leading cause of morbidity and mortality worldwide [2], its pathobiology remains incompletely understood, particularly with respect to nutritional and metabolic modulators.

Among dietary variables, glutamate, a common dietary component widely used as a food additive, has been increasingly recognized for its potential pathogenic effects beyond its role as a food additive. Epidemiological studies have reported associations between plasma glutamate levels and cardiovascular risk and subclinical atherosclerosis [3–5]. However, whether this association reflects a causal role for glutamate in atherogenesis or merely represents an epiphenomenon of underlying metabolic disturbances remains unclear. Importantly, exogenous glutamate from dietary sources does not consistently elevate circulating levels in healthy individuals due to efficient homeostatic control [6], raising the possibility that glutamate may exert local effects within the vascular wall, although this remains hypothetical and requires direct investigation.

To examine whether exogenous glutamate administration is associated with atherosclerotic progression independently of systemic metabolic confounders, we employed the ApoE ⁻ / ⁻ mouse model, a well-established model of hypercholesterolemia and atherosclerosis [7]. By administering exogenous glutamate to mice fed either a normal chow or a high-fat diet, we sought to determine whether glutamate supplementation is associated with exacerbated atherosclerotic progression and, if so, whether this effect requires a pro-atherogenic lipid environment. This experimental design allows us to isolate the effect of exogenous glutamate from endogenous metabolic variations. Of note, in our model, serum glutamate levels remain within the normal range (Fig 2E), enabling us to investigate local vascular effects independent of sustained systemic elevation.

Advances in proteomics technologies have enabled unbiased identification of protein signatures associated with atherosclerotic plaque progression [8], offering new opportunities to uncover molecular candidates that may mediate disease processes. In this study, we applied quantitative proteomics profiling to identify proteins whose expression changes are associated with exogenous glutamate administration. Among the identified candidates, OPN (osteopontin), RAPGEF1 (C3G), and MYL7 were differentially expressed in association with glutamate administration and may serve as candidate molecules for further investigation. OPN is known to participate in inflammatory processes, RAPGEF1 is involved in Rap1 signaling, and MYL7 is a myosin light chain related to smooth muscle function.

Our findings reveal that glutamate administration is associated with exacerbated AS progression when combined with a high-fat diet, despite the absence of sustained elevation in circulating glutamate levels. We identify three hub proteins, including RAPGEF1, OPN, and MYL7, as candidate mediators of these associations. These results provide insights into the associations between exogenous glutamate administration and atherosclerotic progression under experimental conditions and, highlight the need for further investigation to determine the relevance of these findings to human dietary exposures.

## 2. Materials and methods

### 2.1. Animals and experimental design

Male *ApoE* ⁻ / ⁻ mice (n = 40, 42 ~ 48 days old) were purchased from Beijing Weitong Lihua Laboratory Animal Technology Company (Beijing, China). The weight range at the beginning of the experiment was 19 ~ 21 g. *ApoE* ⁻ / ⁻ mice were randomized into four groups: AS-GLU group (n = 10) were fed with high-fat diet (HFD) and intragastric administration glutamate (2 g/kg body weight, twice a day); AS group (n = 10) were fed with HFD and intragastric administration saline placebo; Glu group (n = 10) were fed with regular diet and intragastric administration glutamate (2 g/kg body weight, twice a day). The regular chow diet and high-fat diet (HFD) were purchased from Jiangsu Medicience Biopharmaceutical Co., Ltd. The regular chow diet (MD12031) contained 10 kcal% fat, 70 kcal% carbohydrates, and 20 kcal% protein, while the HFD (MD12015) contained 40 kcal% fat, 43 kcal% carbohydrates, and 17 kcal% protein. Control group (n = 10) were fed with regular diet and intragastric administration saline. Oral gavage was chosen to ensure precise and consistent dosing of glutamate, as food and water intake can be variable, particularly in HFD-fed mice. To control for procedural stress, all groups underwent the same gavage schedule, with control and AS groups receiving saline placebo. No significant differences in final body weight were observed among the four groups (S1 Fig), indicating that the observed differences in atherosclerotic burden were not attributable to generalized metabolic effects such as obesity. Three months after the initial intragastric administration, animals were humanely euthanized in accordance with animal welfare guidelines. Preemptive analgesia was not applied as euthanasia was performed under deep anesthesia without painful procedures. Animals were deeply anesthetized with 3 ~ 4% isoflurane via inhalation until loss of pedal and corneal reflexes, followed by cervical dislocation to confirm death. The aortas were then isolated, stripped of peripheral fat, and dissected along the longitudinal axis. Subsequently, the aortic tissues were either flash-frozen in liquid nitrogen or fixed in 4% formalin and embedded in paraffin for further analysis. For anatomical sites, we selected the aortic root, which is the most commonly used and representative site for quantifying atherosclerotic lesions in ApoE ⁻ / ⁻ mice, as well as the entire aorta for evaluating the overall scope of lesions. Blinded analysis was conducted by two independent researchers using a double-blind method, with grouping information concealed. Regarding the number of sections examined per mouse, five consecutive aortic root sections (10 μm apart) were selected from each mouse, and the average value of lesion area was calculated to reduce individual differences. The animal use protocol was approved by the Ethics Committee of Tianjin Huanhu hospital and was in compliance with the National Institutes of Health Guidelines for the Care and Use of Laboratory Animals.

### 2.2. Histopathological and Immunofluorescence Staining Analysis

Tissues of the aorta were fixed in 4% paraformaldehyde and processed through routine dehydration and paraffin embedding to generate serial sections with a thickness of 5 μm. These sections were subjected to a series of histopathological staining protocols. Hematoxylin and eosin (HE) staining was performed to assess the general architecture of the aortic wall and characterize cellular infiltration patterns. Masson trichrome staining was utilized to examine the distribution of collagen fibers and quantify the extent of fibrosis within the aortic tissue. Additionally, Oil Red O staining was conducted to visualize lipid accumulation and evaluate the atherosclerotic plaque burden in the aortic wall. Following staining, the sections were examined under a light microscope, and representative images were captured for subsequent semi-quantitative analysis. All lesion quantifications were performed in a double-blinded manner by two independent researchers. The quantification procedures and software were described as follows. For the aortic root lesion area, Image J software was used to measure the lesion area and the average value was calculated. The lesion area ratio was expressed as (lesion area/ total intima-media area) × 100%. For the en face aorta analysis, Oil Red O-positive lipid deposition area was quantified using Image J software, and the lesion ratio was calculated as (lipid deposition area/ total aortic surface area) × 100%.

For immunofluorescence staining, paraffin sections were first deparaffinized and underwent antigen retrieval to expose target epitopes. The sections were then incubated overnight at 4°C with primary antibodies targeting CD68 and α-SMA at a dilution of 1:200. After washing with phosphate-buffered saline to remove unbound primary antibodies, the sections were incubated with Alexa Fluor 594-conjugated secondary antibodies at a dilution of 1:500 for one hour at room temperature. Nuclei were counterstained with 4,6-diamidino-2-phenylindole at a concentration of 1:1000. Finally, the stained sections were mounted and observed using a laser scanning confocal microscope. Image J software was employed to conduct quantitative analyses of fluorescence signal intensity and the percentage of positively stained cells.

### 2.3. Biochemical Parameter Analysis

The serum levels of total cholesterol (TC), triglyceride (TG), low-density lipoprotein cholesterol (LDL) and high-density lipoprotein cholesterol (HDL) were determined by Mindray BS-240 VET fully automatic veterinary biochemistry analyzer (Shenzhen Mindray Bio-Medical Electronics Co., Ltd., China) according to the manufacturer's protocols. The concentration of glutamic acid in serum was measured using a glutamic acid assay kit (Solarbio, Beijing, China, BC1585) in accordance with the instructions provided by the manufacturer.

### 2.4. Data-independent acquisition relative quantitative proteomics

The mouse aortic tissue specimens underwent mechanical lysis using an MP FastPrep-24 homogenizer (24 × 2, 6.0 M/S, 60 seconds, twice). Subsequently, lysates were solubilized in SDC buffer (5% SDC, 100 mM Tris-HCl, pH 8.5) followed by DTT incubation at 37°C for 1.5 hours to reduce disulfide bonds. Then, IAA was added to alkylate the reduced cysteine residues, and the reaction was allowed to proceed at room temperature in the dark for 30 minutes. Trypsin was then added to the specimen for protein digestion, which was carried out at 37°C for 15 ~ 18 hours. The resulting peptides were desalted using an MCX desalting column (Omicsolution, OS-MCX-1ML), concentrated via vacuum centrifugation, and reconstituted in 20 μL of 0.1% (v/v) formic acid in water. Peptide concentration was estimated by measuring the UV absorbance at 280 nm. For Data-Independent Acquisition (DIA) experiments, iRT (Calibration Retention Time) calibration peptides were spiked into each specimen.

The peptide specimens were analyzed using an Orbitrap™ Astral™ mass spectrometer (Thermo Scientific) coupled to a Vanquish Neo liquid chromatography system (Thermo Scientific) operating in DIA mode. Precursor Ions were scanned at a mass range of 380 ~ 980 m/z, MS1 resolution was 240000 at 200 m/z, Normalized AGC Target: 500%, Maximum IT: 5 ms. 299 windows were set for DIA mode in MS2 scanning, Isolation Window: 2 m/z, HCD Collision Energy: 25 ev, Normalized AGC Target: 500%, Maximum IT: 3 ms. The acquired DIA data were processed using DIA-NN 1.8.1 software. Protein identification was performed with a 99% confidence threshold, corresponding to a false discovery rate (FDR) of ≤ 1%.

### 2.5. Volcano Plot and Heatmap Analysis

Volcano plots were constructed based on proteomic profiles between the high-fat diet group and the high-fat diet plus glutamic acid group. The x-axis indicated log2-transformed fold change, and the y-axis represented -log10-transformed P-value. Differentially expressed proteins were defined as those meeting the criteria of |log2(fold change)| > 1 and *p* < 0.05. Heatmaps were generated using the identified differentially expressed proteins. The horizontal axis displayed individual samples with distinct colors denoting different experimental groups, while the vertical axis represented differentially expressed proteins. Expression levels of significantly regulated proteins were standardized using the Z-score method and visualized with color gradients in the heatmap.

### 2.6. Gene Ontology and KEGG Analysis

Metascape (v3.5, San Diego, CA, USA) is a powerful tool for exploring the potential biological processes and associated pathways of transcriptome and genome data. The protein sequences of the differentially expressed proteins were locally analyzed using the NCBI BLAST+ client software and InterProScan to identify homologous sequences. Gene Ontology (GO) terms were then mapped, and sequences were annotated using the Blast2GO software. After annotation, the proteins were queried against the Kyoto Encyclopedia of Genes and Genomes (KEGG) database to obtain their KEGG orthology identifiers, which were subsequently mapped to KEGG pathways [9–11]. The functional and pathway terms were extracted for further visualization and analysis using R software.

### 2.7. Putative Signaling Pathways Involving Hub Genes and GO Analysis

GeneMANIA (http://www.genemania.org) is a versatile web-based tool for generating hypotheses about gene function, analyzing gene lists, and prioritizing genes for functional assays. It visualizes relationships between genes and datasets by constructing interactive functional association networks [12]. The identified hub genes were imported into the GeneMANIA database to construct a putative protein-protein interaction network. Subsequently, the genes derived from the network were subjected to Gene Ontology (GO) analysis using the Metascape tool (v3.5, San Diego, CA, USA). The results were processed and visualized using R software.

### 2.8. Western blotting

Aortic tissue lysates were subjected to Western blotting to evaluate the protein expression levels of RAPGEF1, Osteopontin (OPN) and MYL7. The weighed aortic tissue was placed in a 2.0 ml grinding tube containing RIPA lysis buffer (Beyotime Biotechnology, Beijing, China) and grinding beads, and then ground in a 4°C grinder. The ground tissue was placed on ice for lysis for 20 minutes, and then the lysis tube was taken out and mixed up and down. It was then centrifuged at 12,000 rpm for 20 minutes at 4°C in a low-temperature high-speed centrifuge. The supernatant after centrifugation was transferred to a 1.5 ml centrifuge tube, which was the total protein sample. The protein concentration was determined by the BCA method (Beyotime Biotechnology, Beijing, China). Protein samples were aliquoted and stored at −80°C until detection. Fifty micrograms of proteins were separated by 10% SDS-PAGE and then were transferred to PVDF membrane followed by blocking with 5% non-fat dried milk and incubation with primary antibodies (1:1000 for RAPGEF1 and MYL7, 1:750 for OPN, 1:3000 for β-tubulin, respectively) at 4°C overnight. Blots were then incubated with a horseradish peroxidase-conjugated secondary antibody (1:5000) at room temperature for 1 h, then chemiluminescent detection was employed using the ECL system (BIOSHARP, Hefei, China). Images were captured using the Amersham Imager 600 (GE Healthcare Bio-Sciences AB, MA, USA). The used primary antibodies were as follows: RAPGEF1 and MYL7 (HUABIO Technology, Hangzhou, China); OPN (Proteintech, Wuhan, China); β-tubulin (Beijing Zhongshan Golden Bridge Biotech., Beijing, China).

### 2.9. RNA isolation and qRT-PCR analysis

Total RNA was extracted from 30 mg aortic tissue specimens using TRIzol^®^ Reagent (Invitrogen, CA, USA) according to the manufacturer's instructions. Total RNA was reversely transcribed using M-MLV RT Premix (Accurate biology, Changsha, China) following the manufacturer's protocol. Expressions of RNAs were carried out using SYBR-based quantitative real-time PCR with the 7500 real-time PCR system (Applied Biosystems, CA, USA). 18s rRNA was used as the endogenous control to avoid technical and experimental variations among groups. Specific primers were obtained from Sangon Biotech (Shanghai, China). The fold changes in gene expression alongside 18s RNA were determined using 2^﹣∆∆Ct^ method. The primer sequences for qPCR are provided in the supplementary table (S1 Table).

### 2.10. Statistical analysis

All statistical analyses were performed using GraphPad Prism 9.0 (GraphPad Software, Inc., La Jolla, CA, USA). Normality of all data distributions was verified using the Shapiro-Wilk test, and all datasets met the normality assumption (*p* > 0.05), justifying the use of parametric methods.

For terminal single-time-point biochemical indicators measured under the 2 × 2 factorial design, two-way analysis of variance (two-way ANOVA) was used to evaluate the main effects of diet intervention and glutamate intervention, as well as their interaction. Tukey's multiple comparisons test was applied for post-hoc pairwise comparisons to control the family-wise error rate. Grubbs’ test (extreme studentized deviate method) was employed to identify potential outliers, and no outliers were detected or excluded from any dataset. All quantitative data are presented as the mean ± standard deviation (SD). A two-tailed *p*-value < 0.05 was considered statistically significant for all comparisons.

For longitudinal body weight data recorded at Week 0, 4, 8, and 12 (S1 Fig), a three-way repeated-measures ANOVA was applied to fit the hierarchical structure of the 2 × 2 factorial longitudinal dataset. This model includes two fixed between-subject factors (diet intervention and glutamate intervention) and one within-subject repeated factor (time), with individual mice treated as nested biological replicates to account for the intrinsic correlation of repeated measurements from the same animal over time.

### 2.11. Batch Effect Control

To minimize technical variability and eliminate batch effects in the proteomics analysis, a multi-layered strategy was implemented across experimental design and subsequent data processing steps. All proteomic samples were processed and subjected to mass spectrometry analysis in a single experimental batch to abrogate batch effects at the experimental source. For data preprocessing, raw mass spectrometry data were normalized via quantile normalization to establish inter-sample comparability. Potential residual batch effects were further attenuated using the ComBat function embedded in the R sva package (version 3.46.0), a well-validated approach for batch effect correction in high-dimensional omics data. To verify the efficacy of batch effect control, principal component analysis (PCA) was performed on the normalized proteomics data; no evident batch- or processing run-based clustering was observed (S2 Fig), confirming the effective mitigation of technical batch-related variability.

All key experimental protocols described in this study have been deposited in protocols.io and are publicly accessible via the following DOI: https://dx.doi.org/10.17504/protocols.io.yxmvm8b85g3p/v1.

## 3. Results

### 3.1. Glutamate administration is associated with exacerbated formation of AS plaques in ApoE^−/−^mice

**Fig 1:**
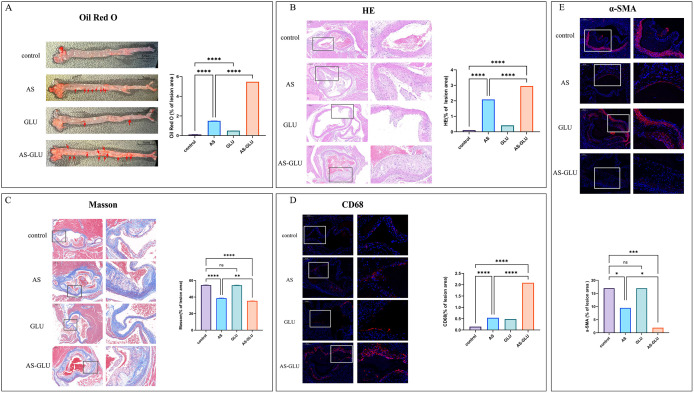
Effects of glutamate administration on atherosclerotic plaque formation, intima thickness and plaque composition in ApoE ⁻ / ⁻ mice. A, Oil red O staining showing of aortic en face preparations. Red stained areas indicate atherosclerotic plaque. Red arrows indicate visible plaque areas. The graph on the right shows quantification of plaque area as percentage of total aortic surface; B, H&E staining showing general morphology and intima thickness. The graph on the right shows quantification of intima thickness; C, Masson’s trichrome staining showing collagen deposition (blue) within the plaque. The graph on the right shows quantification of collagen volume fraction; D, Immunofluorescence staining for CD68 (a macrophage marker). Red fluorescence indicates macrophage infiltration within the plaque. The graph indicates macrophage infiltration with plaque. The graph on the right shows quantification of CD68 positive area; E, Immunofluorescene staining for α-SMA (vascular smooth muscle cell marker). Red fluorescence indicates smooth muscle cell within the plaque. The graph on the right shows quantification of α-SMA positive area. Data are presented as mean ± SD. Statistical analysis was performed using two-way ANOVA with Tukey’s post-hoc test, (n = 3 for each group). *p < 0.05, **p < 0.01, ns *p* > 0.05.

In *ApoE*^−/−^ mice fed a high-fat diet for three months, pronounced atherosclerotic (AS) plaques were histologically confirmed in the aortic specimens (Fig 1A), contrasting with the intact vascular morphology observed in normal control mice (Fig 1A), indicating the successful establishment of the AS model in this study. Notably, chronic administration of glutamate in conjunction with a high-lipid diet was associated with a greater AS burden, as evidenced by the increased plaque size revealed in the oil red O staining (Fig 1A). Quantitative analysis showed that the plaque area in the AS-GLU group was significantly larger compared to the AS group (*p* < 0.05). Additionally, H&E staining demonstrated that glutamate administration with a high-lipid diet markedly increased intima thickness in the AS-GLU group compared to the AS group (Fig 1B). In the Control group, the aortic wall structure was intact, with uniform intima-media thickness, regular vascular lumen morphology, and almost no lipid deposition; no significant abnormalities were observed. While in the AS group, the intima-media was significantly thickened, with localized plaque-like structures protruding into the lumen, resulting in mild luminal stenosis. Within the plaques, extensive inflammatory cell infiltration was observed, forming distinct inflammatory foci, and aggregates of foam cells were seen in some areas. In the GLU group, the degree of intima-media thickening was milder compared to the AS group, and inflammatory cell infiltration was significantly reduced, with only a few inflammatory cells observed locally beneath the intima and no obvious foam cell formation. In the AS-GLU group, the intima-media was markedly thickened, with large plaques protruding into the lumen, causing severe luminal stenosis. The intima-media structure was disrupted, with the normal layered architecture lost, and needle-like or cleft-like vacuoles representing cholesterol crystals were observed. At the plaque edge, as well as at the junction of the fibrous cap and the media, extensive inflammatory cell infiltration was present, creating well-defined inflammatory bands. These findings suggest that glutamate acts as a lipid co-factor associated with AS progression.

### 3.2. Detection of Atherosclerosis Biomarkers

To evaluate extracellular matrix remodeling and plaque stability, Masson trichrome staining was performed to quantify the collagen volume fraction (CVF) in aortic sections (Fig 1C). As shown in figure, the control group exhibited abundant collagen deposition. The GLU group showed a comparable CVF (*p* > 0.05 *vs.* control), indicating that glutamate alone is not associated with direct changes in vascular collagen metabolism under physiological conditions. In contrast, the AS group displayed a significant reduction in CVF (*p* < 0.05 *vs.* control), confirming successful establishment of atherosclerotic plaques with characteristic collagen degradation. Notably, compared with the AS group, the AS-GLU group exhibited a further marked decrease in CVF (*p* < 0.05), demonstrating that glutamate intervention is associated with exacerbated collagen loss in the context of atherosclerosis (Fig 1C). These results suggest that while glutamate itself does not directly impair vascular collagen integrity, it is associated with enhanced atherosclerosis-induced extracellular matrix degradation.

Compared with the control group, the AS group exhibited a significantly increased CD68-positive macrophage infiltration (*p* < 0.05) (Fig 1D) and a marked reduction in α-SMA-positive vascular smooth muscle cells (*p* < 0.05) (Fig 1E), suggesting successful establishment of the atherosclerotic lesion model, characterized by enhanced intraplaque inflammation and reduced plaque stability. Notably, compared with the AS group, the AS-Glu group showed a further significant increase in the CD68-positive area (*p* < 0.05) and a more pronounced decrease in the α-SMA-positive area (*p* < 0.05). In contrast, the Glu group exhibited mildly elevated CD68 expression (Fig 1D) and slight reductions in α-SMA compared with the control group (Fig 1E). Collectively, these results indicate that glutamate intervention is associated with exacerbated atherosclerotic lesion progression and features of plaque instability, including increased macrophage infiltration and reduced vascular smooth muscle cell content.

### 3.3. Serum levels of lipids and glutamic acid in mice

**Fig 2:**
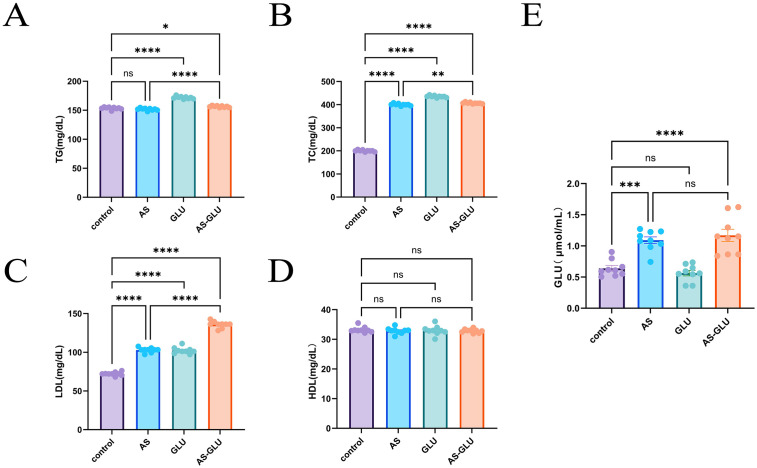
Effects of glutamate on serum lipid profiles and circulating glutamate levels in ApoE ⁻ / ⁻ mice. A, Serum levels of triglycerides (TG); B, Serum total cholesterol (TC) level; C, Serum low-density lipoprotein cholesterol (LDL-C) levels; D, Serum high-density lipoprotein cholesterol (HDL-C) levels; E, Fasting serum glutamate concentrations (n = 9 per group). Data are presented as mean ± SD. Statistical analysis was performed using two‑way ANOVA with Tukey’s post‑hoc test. *p < 0.05，**p < 0.01，****p < 0.0001.

To evaluate the impact of glutamate intervention on systemic lipid metabolism and glutamate homeostasis, serum lipid profiles and glutamate concentrations were measured in each group, with the results presented in Fig 2. For lipid profiles, no significant difference in serum triglyceride (TG) levels was observed between the Control group and the AS group (*p* > 0.05). Notably, the Glu group exhibited the highest serum TG levels, and the AS-GLU group showed TG levels comparable to those of the Glu group, both of which were significantly higher in the AS group (*p* < 0.05) (Fig 2A). Serum total cholesterol (TC) levels were significantly elevated in the AS, Glu, and AS-GLU groups compared to the Control group. Specifically, TC levels in the AS-GLU group were similar to those in the Glu group and were significantly higher than those in the AS group (*p* < 0.05) (Fig 2B). For low-density lipoprotein cholesterol (LDL-C), both the AS group and the Glu group exhibited similarly elevated levels, which were significantly higher than those in the Control group. The AS-GLU group displayed the highest LDL-C levels, which were significantly increased compared to the Control group (*p* < 0.05) and also significantly exceeded those in both the AS and Glu groups (*p* < 0.05) (Fig 2C). Serum high-density lipoprotein cholesterol (HDL-C) levels showed no statistically significant differences among all experimental groups (*p* > 0.05) (Fig 2D).

For serum glutamate levels, chronic intragastric administration of glutamate did not significantly alter serum glutamate levels in the Glu group compared with the control group (*p* > 0.05). The baseline serum glutamate level in the AS group was significantly elevated relative to the control group (*p* < 0.05). The AS-GLU group showed a numerical increase in serum glutamate levels compared with the AS group, but this difference did not reach statistical significance (*p* > 0.05) (Fig 2E).

### 3.4. Body weight changes

Body weight was monitored at weeks 0, 4, 8, and 12 in all four groups (S1 Fig). Three-way repeated-measures ANOVA showed no significant differences in body weight among the Control, AS, Glu, and AS-GLU groups at any time point (all *p* > 0.05). These results confirm that the observed differences in atherosclerotic burden were not attributable to generalized obesity or weight-related metabolic effects.

### 3.5. Identifications of Differentially Expressed Genes (DEGs)

**Fig 3:**
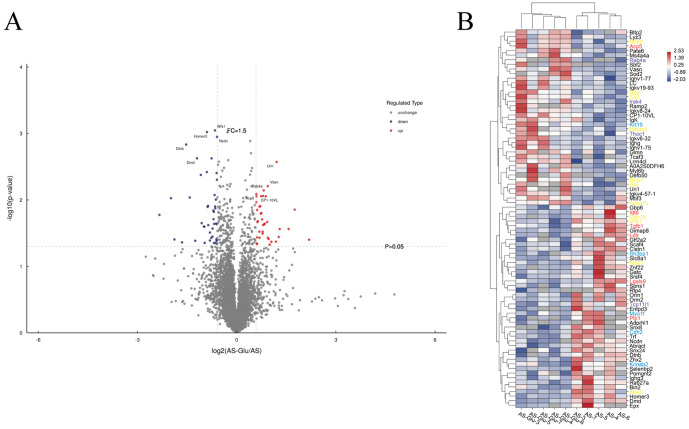
Identification of differentially expressed proteins (DEPs) between comparison groups. A, Volcano plot showing the distribution of differentially expressed proteins based on fold change (FC) and statistical significance (p-value, T-test). Proteins with significant down-regulation (FC < 0.67, *p* < 0.05) are labeled in blue, while proteins with significant up-regulation (FC > 1.5, *p* < 0.05) are labeled in red. Proteins with no significant differences are shown in grey. The top five most significantly up- and down-regulated proteins are highlighted (n = 5); B, Heatmap illustrating the hierarchical clustering of differentially expressed proteins across comparison groups. The heatmap provides a visual representation of the relative expression levels of proteins, grouped and categorized based on their expression patterns (n = 5).

To identify significant differences in protein expression between the comparison groups, a volcano plot was constructed based on two key parameters: fold change (FC) and statistical significance (*p*-value, T-test) (Fig 3A). In the volcano plot, 44 significantly down-regulated proteins (labeled in blue, FC < 0.67 and *p* < 0.05) and 38 significantly up-regulated proteins were recorded (labeled in red, FC > 1.5 and *p* < 0.05). Proteins with no significant difference were depicted in grey. To further highlight the most prominent changes, five most dysregulated proteins in each direction were explicitly annotated.

Additionally, the differentially expressed proteins were grouped and categorized based on their expression patterns, and their relative expression levels were visualized using a heatmap (Fig 3B). This heatmap provides a comprehensive overview of the protein expression profiles across the comparison groups, facilitating the identification of potential key regulatory proteins involved in the biological processes under investigation. This analysis not only underscores the distinct protein expression changes between the groups but also provides a foundation for further functional studies to elucidate the roles of these differentially expressed proteins in the underlying biological mechanisms.

### 3.6. Functional Enrichment Analysis

**Fig 4:**
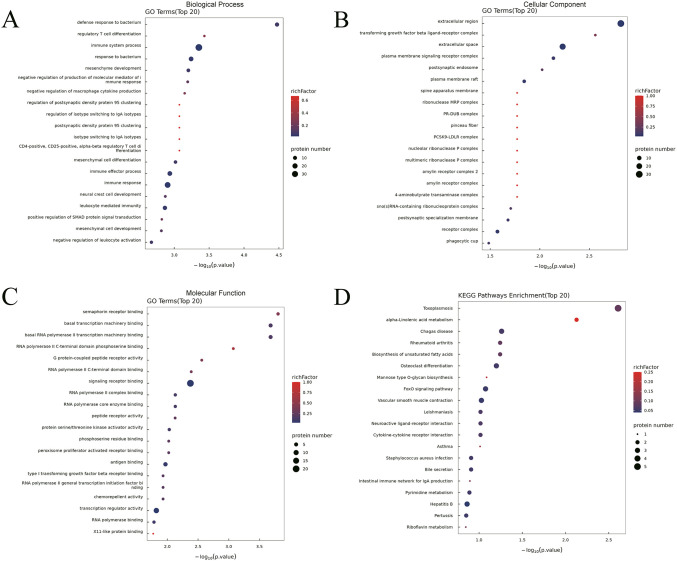
Functional enrichment analysis of differentially expressed proteins (DEPs). A, Biological processes enriched among DEPs, highlighting immune-related processes such as defense response to bacterium and regulatory T cell differentiation, as well as developmental and regulatory processes (n = 5); B, Cellular components associated with DEPs, including extracellular regions, plasma membrane structures, and multimeric complexes (n = 5); C, Molecular functions of DEPs, emphasizing receptor binding, transcriptional regulation, and enzymatic activities (n = 5); D, KEGG pathway enrichment of DEPs, showing the top 20 enriched pathways related to immune responses, metabolism, and disease pathogenesis (n = 5).

Functional enrichment analysis was performed to identify the biological roles of differentially expressed proteins (DEPs) showed in Fig 4 (All enrichment analyses are based on differentially expressed proteins between the AS group and the AS-GLU group). By comparing all DEPs against the reference species’ annotated proteins with Gene Ontology (GO) functions, the enriched functional categories were determined, and the significance of enrichment was assessed using Fisher's Exact Test (*p*-value < 0.05). Bubble plots were used to visualize the enriched GO entries across the three major GO categories: biological processes, cellular components, and molecular functions.

In the biological processes category, the DEPs were significantly enriched in immune-related terms such as defense response to bacterium, regulatory T cell differentiation, and leukocyte-mediated immunity, as well as developmental and regulatory processes including neural crest cell development and negative regulation of leukocyte activation (Fig 4A). For cellular components, the DEPs were predominantly localized in the extracellular region, plasma membrane, and transforming growth factor beta ligand-receptor complex, along with multimeric complexes such as the ribonuclease P complex and amylin receptor complex (Fig 4B). In terms of molecular functions, the DEPs were associated with receptor binding (e.g., G protein-coupled peptide receptor activity), transcriptional regulation (e.g., RNA polymerase II binding), and enzymatic activity (e.g., protein serine/threonine kinase activator activity) (Fig 4C). Additionally, KEGG pathway analysis revealed enrichment in immune-related pathways such as Rheumatoid arthritis and Staphylococcus aureus infection, metabolic pathways including Biosynthesis of unsaturated fatty acids, and disease-related pathways such as Chagas disease and Hepatitis B (Fig 4D).

### 3.7. Protein-Protein Interaction (PPI) Network Construction and Hub Gene Analysis

**Fig 5:**
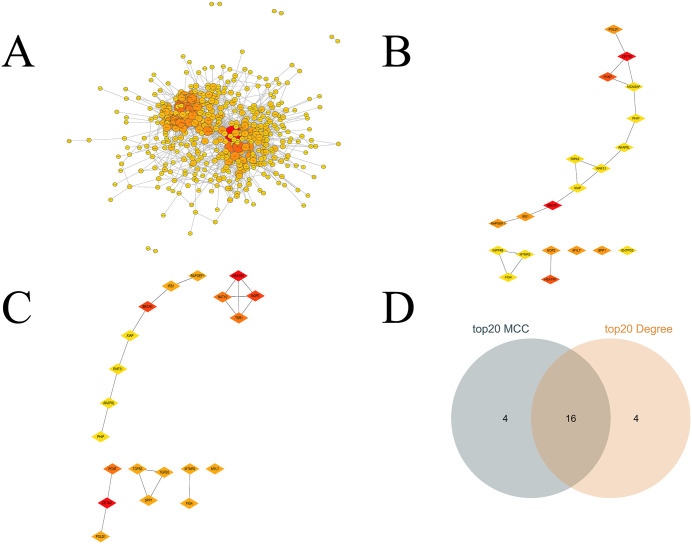
Protein-protein interaction (PPI) network analysis of differentially expressed proteins (DEPs). A, Visualization of the PPI network, where nodes represent proteins and edges represent interactions. Node size and color indicate centrality scores, highlighting highly connected hub proteins; B, Top 20 hub proteins regulating the expression of RAPGEF1, OPN, and MYL7 and their associated signaling pathways identified based on Degree, representing key regulatory proteins within the network (n = 5); C, Top 20 hub proteins identified based on Maximal Clique Centrality (MCC), representing key regulatory proteins within the network (n = 5); D, Intersection of the top 20 hub proteins from Degree and MCC analyses, identifying 16 key target genes in the PPI network.

To further explore the functional relationships among the differentially expressed proteins (DEPs), a protein-protein interaction (PPI) network was constructed and analyzed. The overall structure of the PPI network is shown in Fig 5A, where nodes represent proteins and edges represent interactions. The size and color of the nodes correspond to their centrality scores, emphasizing the importance of highly connected hub proteins. Using the CytoHubba plugin in Cytoscape [13,14], the top 20 hub proteins were identified based on two centrality measures: Degree (Fig 5B) and Maximal Clique Centrality (MCC) (Fig 5C). By taking the intersection of these results, 16 key target genes were identified (Fig 5D and Table 1), including *CETN2*, *HEATR1*, *BECN1*, *NOP2*, *PCNT*, *SPP1(OPN)*, *IRS1*, *RAPGEF1*, *POLD1*, *FIG4,MTMR2*, *MYL7*, *AKAP8L*, *PHIP*, *RNF31*, and *XIAP*. Among these 16 candidates, OPN (SPP1), RAPGEF1, and MYL7 were selected for further validation based on their documented roles in vascular biology: OPN in inflammation and foam cell formation, RAPGEF1 in integrin signaling and endothelial function, and MYL7 in smooth muscle contraction and cytoskeletal regulation, which are processes centrally involved in atherosclerosis pathogenesis (S3 Fig).

**Table 1:** Top 20 hub genes identified by MCC and Degree algorithms and their overlapping genes.

MCC	Degree	Both
CETN2	BECN1	CETN2
HEATR1	CETN2	HEATR1
BECN1	PCNT	BECN1
NOP2	HEATR1	NOP2
PCNT	SPP1	PCNT
NAT10	IRS1	SPP1
TSR1	RAPGEF1	IRS1
SPP1	POLD1	RAPGEF1
IRS1	NOP2	POLD1
RAPGEF1	MYL7	FIG4
POLD1	AKAP8L	MTMR2
FIG4	PHIP	MYL7
MTMR2	RNF31	AKAP8L
MYL7	XIAP	PHIP
TGFB3	MCM3AP	RNF31
TGFB2	RIPK2	XIAP
AKAP8L	FIG4	–
PHIP	MTMR2	–
RNF31	ENTPD3	–
XIAP	INPP4B	–

### 3.8. Construction of Putative Hub Gene Protein-Protein Interaction Network and GO Analysis

**Fig 6:**
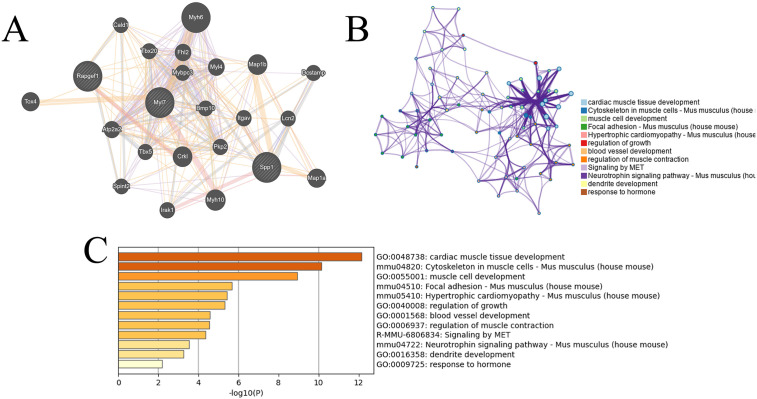
Pathway and functional enrichment analysis of the protein-protein interaction (PPI) network in atherosclerosis (AS). A, Visualization of the PPI network constructed using the GeneMania tool, consisting of 23 genes (including central genes OPN, RAPGEF1, and MYL7) and 175 interaction links; B, Enriched Gene Ontology (GO) terms associated with the 23 genes, highlighting processes such as muscle cell development, cytoskeleton in muscle cells, and tissue development; C, Enriched KEGG pathways, including Focal adhesion, Hypertrophic cardiomyopathy, and blood vessel development, indicating the involvement of these genes in muscle function, cellular adhesion, and vascular biology.

Using the GeneMania tool, we constructed a putative protein-protein interaction (PPI) network consisting of 23 genes, including central genes of OPN, RAPGEF1, and MYL7. The PPI network contains a total of 175 links (Fig 6A). Functional enrichment analysis revealed that these 23 genes were significantly associated with biological processes and pathways related to muscle cell development, cytoskeleton in muscle cells, and tissue development (Figs 6B and C). Additionally, enriched pathways included Focal adhesion, Hypertrophic cardiomyopathy, and blood vessel development. These findings highlight the potential involvement of these genes in muscle function, cellular adhesion, and vascular biology.

### 3.9. Validation of Proteomics Findings by Western blot and qPCR

**Fig 7:**
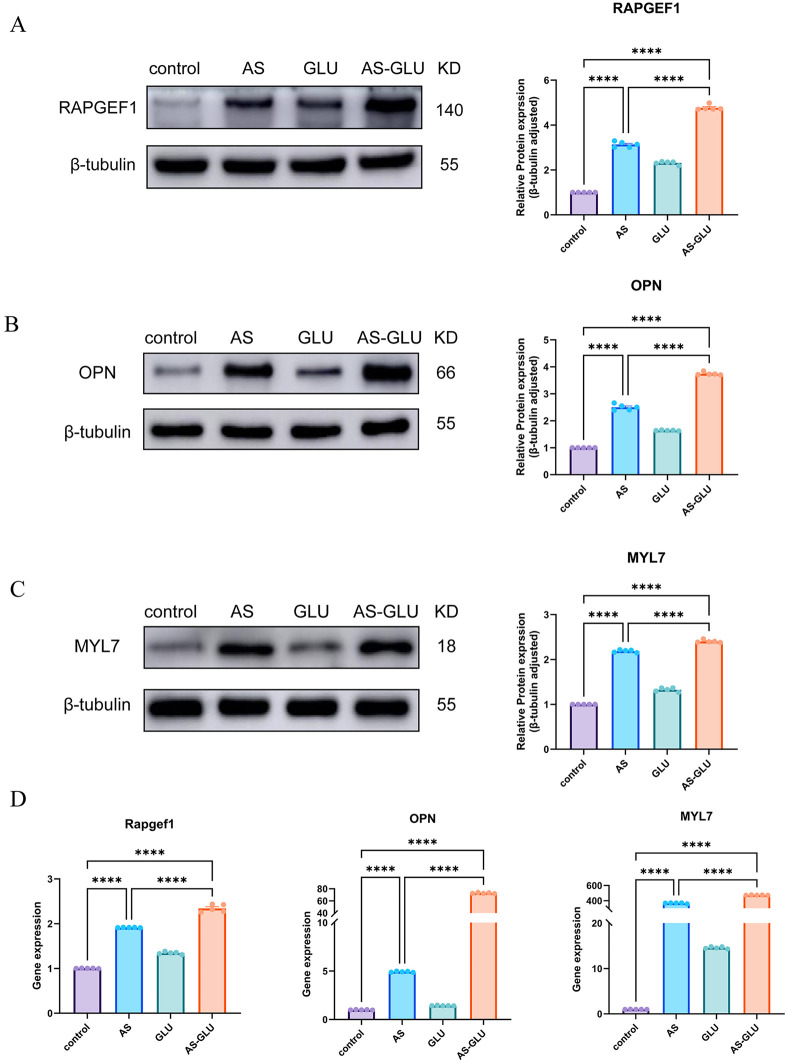
Validation of proteomics findings by Western blot and qPCR. A, B and C show the Western blot results for the proteins RAPGEF1, OPN and MYL7 on the left, with the corresponding grayscale value bar graphs shown on the right (biological replicates are 3 and technical replicates are 3); D displays the relative expression levels of the three genes (RAPGEF1, OPN and MYL7) measured by qPCR (biological replicates are 3 and technical replicates are 3). Significance levels are indicated as follows: *p < 0.05, ***p < 0.001.

To validate the results of our proteomic analysis, we performed Western blot and quantitative real-time PCR (qPCR) to assess the expression levels of the central genes *RAPGEF1*, *OPN*, and *MYL7* in aortic tissues from different experimental groups: normal control, AS group, Glu group, and AS-GLU group. Western blot analysis revealed significant differences in the expression levels of *RAPGEF1* (Fig 7A), *OPN* (Fig 7B), and *MYL7* (Fig 7C) across the groups. Specifically, the AS-GLU group exhibited markedly higher protein expression levels compared to the other groups, consistent with our proteomic sequencing results. These findings further support the potential role of these proteins in glutamate-mediated AS pathogenesis.

qPCR analysis further confirmed the differential expression of *RAPGEF1*, *OPN* and *MYL7* at the mRNA level (Fig 7D). In mouse aortic samples, the expression levels of these genes were significantly higher in the AS-GLU group compared to the normal control, AS group, and Glu group (*p* < 0.05 for all comparisons). These results are consistent with the trends observed in the proteomic and Western blot analyses, reinforcing the reliability of our findings. Together, these validation experiments confirm the differential expression of *RAPGEF1*, *OPN* and *MYL7* in AS progression, particularly under glutamate exposure, and highlight their potential significance in the molecular mechanisms underlying glutamate-induced AS.

## 4. Discussion

In the present study, we used data-independent acquisition (DIA) quantitative proteomic profiling to examine the association between oral glutamate administration and atherosclerotic progression in ApoE^-/-^ mice, revealing molecular signatures that correlate with disease progression. Through bioinformatics analysis including PPI network construction, GO enrichment and KEGG pathway analysis, we identified three hub proteins, OPN, RAPGEF1, and MYL7, whose expression levels were altered in association with glutamate treatment. Further bioinformatics analyses suggested potential biological processes and metabolic pathways that may involve these proteins, providing candidate molecules associated with glutamate administration and atherosclerotic progression for future mechanistic investigation.

In this study, we observed that chronic oral glutamate administration did not significantly elevate fasting serum glutamate levels in healthy mice (Glu group), confirming the robust homeostatic regulation of systemic glutamate under physiological conditions. In contrast, atherosclerotic mice (AS group) exhibited significantly elevated baseline serum glutamate levels, indicating that the disease state itself is associated with disrupted glutamate homeostasis. Notably, although the AS-GLU group showed a numerical increase compared with the AS group, this difference did not reach statistical significance. These findings suggest that the pro-atherogenic changes associated with exogenous glutamate are not primarily driven by a simple dose-dependent increase in systemic concentration. Whether local glutamate accumulation within the vascular wall occurs remains a hypothesis that requires direct measurement of tissue glutamate levels. These observations, suggest that the effects of glutamate administration may depend on the underlying metabolic and atherosclerotic milieu rather than on sustained systemic glutamate elevation alone.

Regarding the cellular context of the observed protein changes, our proteomic analysis was performed on whole aortic tissue and therefore cannot identify the specific cell types responsible for the differential expression of RAPGEF1, OPN, and MYL7. Previous studies have reported that glutamate signaling can influence endothelial cells, vascular smooth muscle cells, and macrophages under certain experimental conditions [3,15,16]. In addition, our histological analyses revealed increased CD68-positive macrophage infiltration and reduced α-SMA-positive VSMC area in the AS-GLU group. Although these observations suggest potential involvement of multiple vascular cell populations, the present study does not allow definitive conclusions regarding cell-specific mechanisms. Future studies employing single-cell RNA sequencing, spatial transcriptomics, or cell-type-specific knockout models will be required to determine the cell origins and functional significance of the observed molecular change (RAPGEF1, OPN, and MYL7).

Glutamate supplementation was used in this study as an experimental intervention to explore the potential impact of excessive glutamate exposure on atherosclerotic progression. However, the administered dose (4g/kg/day) substantially exceeds typical human dietary intake, and therefore the translational relevance of this exposure level should be interpreted with caution. The experimental design, which included glutamate supplementation in both healthy and atherosclerotic backgrounds, allowed us to compare the effects of exogenous glutamate under different metabolic backgrounds. Nevertheless, because serum glutamate levels were not significantly elevated in the AS-GLU group compared to the AS group, and glutamate administration also altered serum lipid profiles, the relative contributions of direct vascular effects and indirect metabolic effects could not be distinguished. Future dose-response studies employing physiologically relevant exposure levels will be necessary to clarify the translational implications of these findings.

SPP1-encoded osteopontin (OPN), an arginine-glycine-aspartate-containing acidic phosphoprotein, has multifaceted functionalities including cell adhesion, chemotactic modulation and calcium-binding properties [17]. Upregulation of OPN in atherosclerotic lesions has been associated with pro-inflammatory and pro-calcification roles, although some studies suggest it may negatively regulate vascular calcification [18]. Previous studies have implicated that OPN orchestrates macrophage recruitment and foam cell formation via integrin-mediated signaling and CD44 receptors [19,20], supporting its previously reported association with plaque progression and vascular inflammation. Notably, our KEGG analysis identified OPN-associated pathways such as “ECM-receptor interaction” and “Toll-like receptor signaling pathway”, these pathways collectively coordinate endothelial dysfunction [21] and extracellular matrix degradation, which are the key features of advanced atherosclerosis [22].

RAPGEF1 (also known as C3G), a guanine nucleotide exchange factor (GEF) for Rap1 GTPase, serves as a critical modulator of vascular morphogenesis and Ras superfamily signaling through Rap1-mediated integrin activation [23,24]. Its enrichment in the “cAMP signaling” pathway suggests a regulatory role in endothelial barrier integrity [25]. Recent evidence indicates that Rap1 activation attenuates vascular inflammation by stabilizing endothelial junctions [26]; however, the potential relationship between glutamate signaling and RAPGEF1 remains unclear. Although previous studies have reported interactions between glutamate receptor signaling and Ras/ERK-related pathways in several biological systems [27,28], none of the components of this signaling cascade were directly assessed in the present study. In addition, RAPGEF1 has been implicated in signaling processes involved in vascular remodeling and cellular responses to metabolic stress [27,28]. Therefore, the potential involvement of RAPGEF1-Ras/ERK signaling in glutamate-associated atherosclerosis remains speculative and requires future experimental validation.

MYL7 encodes a myosin light chain predominantly expressed in cardiovascular tissue and skeletal muscle, involved in the regulation of cardiac development and contraction, especially in embryonic cardiogenesis [29]. GO analysis further linked MYL7 to “leukocyte transendothelial migration” and “calcium ion binding”, supporting its potential involvement in neointima formation. The biological role of MYL7 in atherosclerosis remains poorly understood. Existing studies mainly link MYL7 to cardiovascular development, contractile function, and cytoskeletal regulation. Although our enrichment analysis suggests potential associations with vascular remodeling-related pathways, the mechanistic significance of MYL7 in atherosclerotic plaque development remains to be determined.

Compared with OPN, the role of MYL7 in atherosclerosis remains largely unexplored. Although MYL7 expression was increased in glutamate-treated atherosclerotic mice, the biological significance of this finding remains unclear. Additional studies are required to determine whether MYL7 contributes directly to vascular remodeling and plaque progression or simply reflects secondary changes associated with disease severity.

Taken together, our findings demonstrate that chronic glutamate administration was associated with aggravated atherosclerotic lesion development and altered expression of RAPGEF1, OPN, and MYL7 in ApoE ⁻ / ⁻ mice fed a high-fat diet. However, the present study does not establish causal relationships among these observations. RAPGEF1, OPN, and MYL7 should therefore be considered candidate molecules associated with glutamate administration and disease progression, and their mechanistic roles require further investigation.

### Limitations

While our findings offer insights into the association between glutamate and atherosclerotic progression, several critical limitations must be acknowledged. First, despite chronic oral glutamate administration, serum glutamate levels in the AS-GLU group were not significantly elevated compared with the AS group alone (Fig 2E). Therefore, the mechanism by which exogenous glutamate exerts its pro-atherogenic effects remains unclear; the proposed local vascular accumulation is a hypothesis that requires direct confirmation via tissue glutamate measurements, which were not performed in this study. Second, ApoE ⁻ / ⁻ mice on a normal diet cleared exogenous glutamate normally (Glu group, Fig 2E), indicating that impaired glutamate clearance is not a baseline feature of this model. This finding contradicts our earlier speculation (now removed from the Introduction) that ApoE ⁻ / ⁻ mice recapitulate a clearance defect. Third, glutamate administration significantly worsened the serum lipid profile, including elevated total cholesterol, triglycerides, and LDL-cholesterol (Figs 2A-C). These lipid changes may independently or synergistically contribute to the observed plaque exacerbation, and we cannot distinguish direct vascular effects of glutamate from indirect effects mediated by altered lipid metabolism. Fourth, the identification of RAPGEF1, OPN, and MYL7 as candidate mediators is based on association only; causal roles have not been established. Future studies employing genetic knockdown or pharmacological inhibition are needed to test whether these proteins directly mediate the effects of glutamate on atherosclerosis. Finally, our proteomic analysis was performed on whole aortic tissue, which does not resolve cell-type-specific contributions. Single-cell RNA sequencing or spatial transcriptomics would be required to determine whether the observed protein changes originate from endothelial cells, smooth muscle cells, macrophages, or other vascular cell types.

## 5. Conclusion

In this investigation, we used proteomic analysis, functional enrichment and experimental validation to examine the association between oral glutamate administration and atherosclerosis progression in ApoE ⁻ / ⁻ mice. Our findings show that glutamate treatment, in the context of a high-fat diet, is associated with exacerbated atherosclerotic plaque formation and increased expression of three proteins: RAPGEF1, OPN, and MYL7. These proteins represent candidate mediators of the observed associations, but their causal roles in glutamate-exacerbated atherosclerosis remain to be established. Notably, despite chronic glutamate administration, serum glutamate levels were not significantly elevated in the AS-GLU group compared to the AS group alone, and glutamate worsened serum lipid profiles (TC, TG, LDL-C). Therefore, we cannot distinguish direct vascular effects of glutamate from indirect effects mediated by altered lipid metabolism. Future studies employing tissue glutamate measurements, cell-type-specific knockout models, and causal inference approaches (e.g., Mendelian randomization or pharmacological inhibition) are needed to determine whether RAPGEF1, OPN, or MYL7 directly mediate the pro-atherogenic effects of glutamate and to elucidate the underlying mechanisms. The integration of these findings with existing knowledge on vascular pathophysiology suggest an associations between excessive glutamate exposure, altered lipid metabolism, and aggravated atherosclerotic progression under experimental conditions. All authors agree to publish.

## Supporting information

S1 Fig Body weight changes in ApoE ⁻ / ⁻ mice over the 12-week experimental period. Body weight was monitored at weeks 0, 4, 8, and 12 in the four groups: Control, AS, GLU, and AS-GLU. Data are presented as mean body weight (g). No significant differences in body weight were observed among the four groups at any time point (*p* > 0.05 for all comparisons, three-way repeated-measures ANOVA with Tukey’s post‑hoc test).
(JPG)

S2 Fig Principal component analysis (PCA) verifing the efficacy of batch effect control. (PNG)

S3 Fig Workflow for the identification of hub proteins RAPGEF1, OPN, and MYL7. Schematic diagram illustrating the sequential bioinformatics strategy used to identify the three hub proteins (RAPGEF1, OPN, and MYL7) from proteomic data. First, differentially expressed proteins (DEPs) between the AS and AS-GLU groups were identified based on criteria of |log₂(fold change)| > 1 and *p* < 0.05. Second, a protein-protein interaction (PPI) network was constructed using the DEPs. Third, hub proteins were ranked using two centrality algorithms, Degree and Maximal Clique Centrality (MCC), implemented in the CytoHubba plugin. Fourth, the intersection of the top 20 proteins from both algorithms yielded 16 candidate hub genes. Finally, among these candidates, OPN, RAPGEF1, and MYL7 were selected for further validation based on their documented roles in vascular inflammation, integrin signaling, and smooth muscle function, processes centrally involved in atherosclerosis pathogenesis.
(PNG)

S1 Table Original results of qPCR. (XLSX)

S2 Table Result of serum glutamate levels. (XLSX)

S3 Table Original result of data-independent acquisition relative quantitative proteomics. (XLSX)

S1 File Raw images of Western blot showing the expression of RAPGEF1, OPN and MYL7. (PDF)
