# Supplementary figures and images for: Glutamate administration is associated with aggravated atherosclerosis and altered expression of RAPGEF1, OPN, and MYL7 in High-Fat Diet-Fed ApoE⁻/⁻ Mice
Source: PLoS One. 2026 Jul 28;21(7):e0354719. doi: 10.1371/journal.pone.0354719 (PMC13411909; doi:10.1371/journal.pone.0354719)

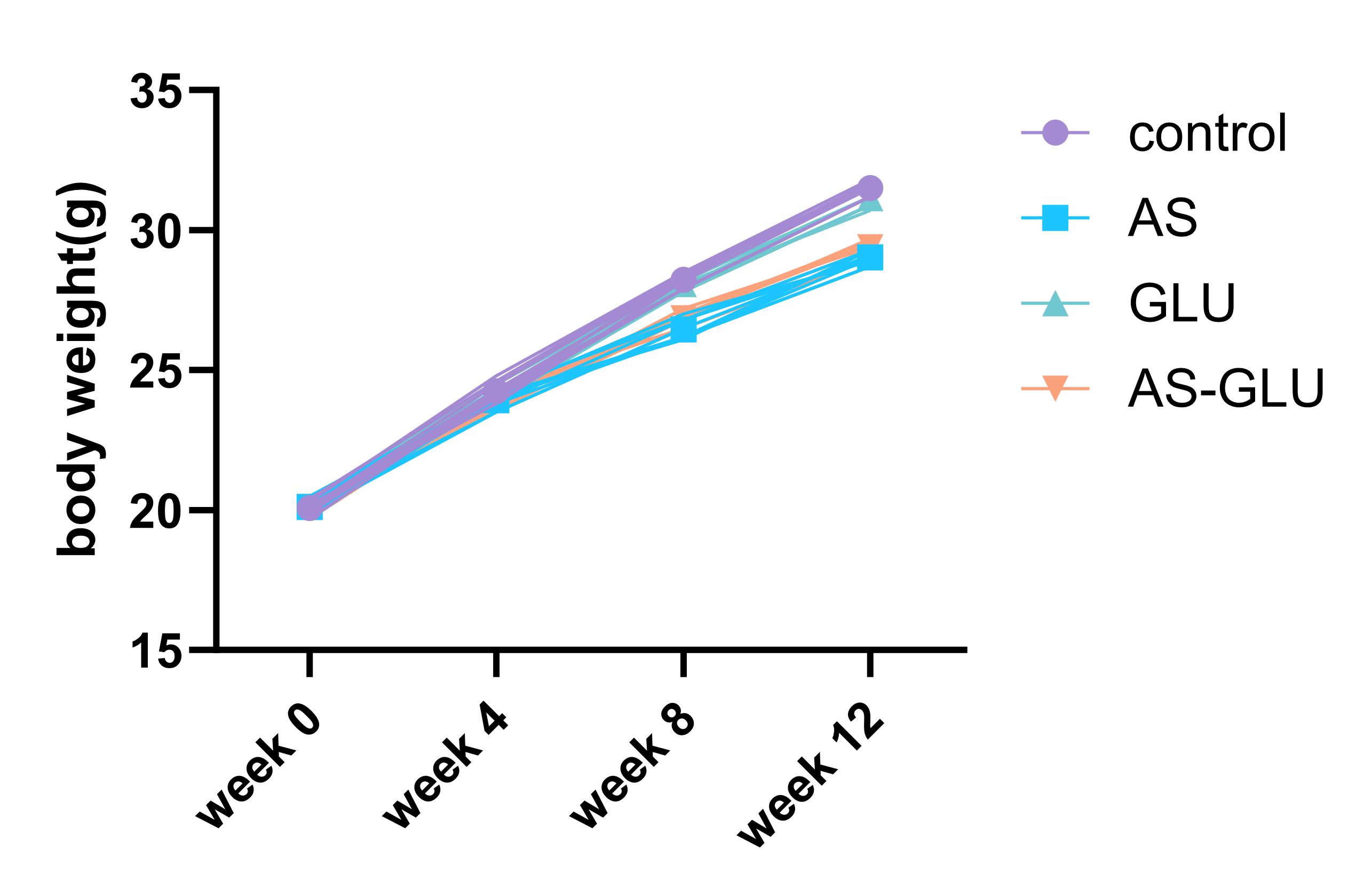

Supplement: S1 Fig — Body weight was monitored at weeks 0, 4, 8, and 12 in the four groups: Control, AS, GLU, and AS-GLU. Data are presented as mean body weight (g). No significant differences in body weight were observed among the four groups at any time point (p > 0.05 for all comparisons, three-way repeated-measures ANOVA with Tukey’s post‑hoc test). (JPG) [file pone.0354719.s001.jpg]

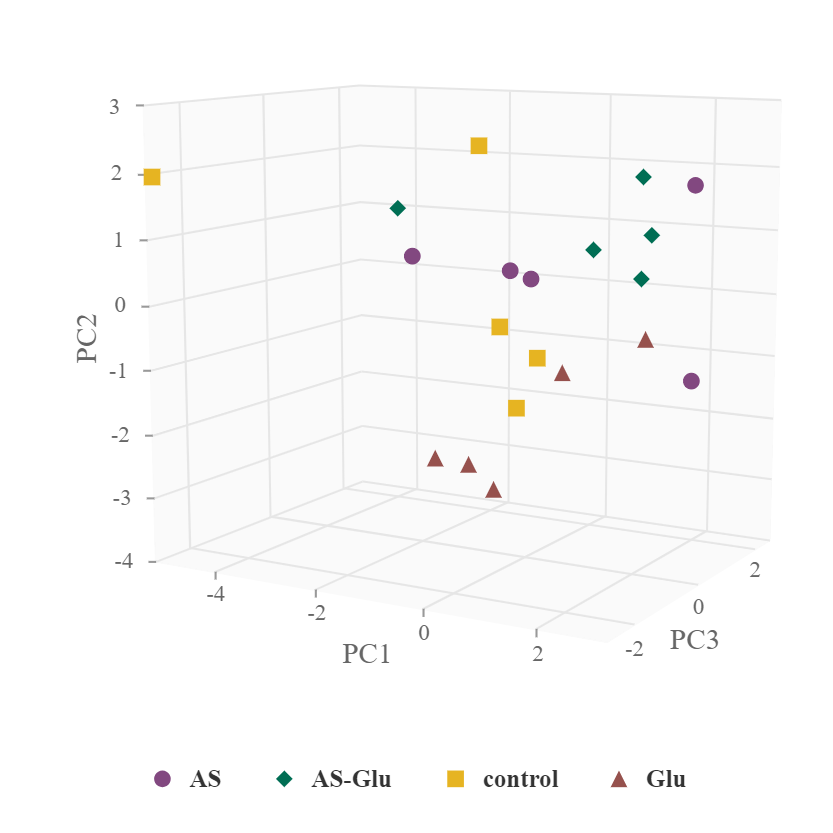

Supplement: S2 Fig — (PNG) [file pone.0354719.s002.png]

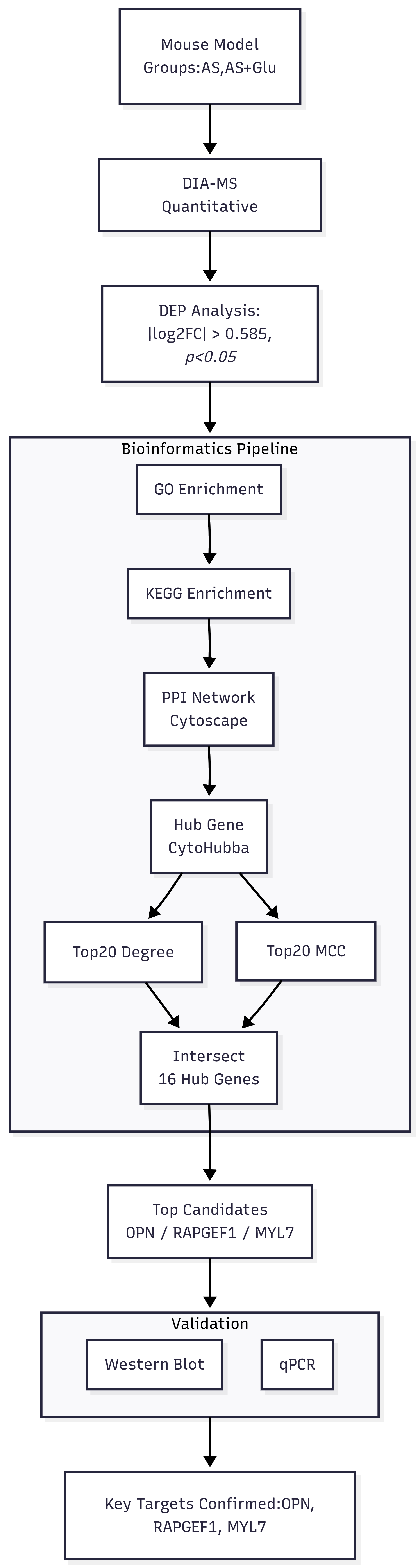

Supplement: S3 Fig — Schematic diagram illustrating the sequential bioinformatics strategy used to identify the three hub proteins (RAPGEF1, OPN, and MYL7) from proteomic data. First, differentially expressed proteins (DEPs) between the AS and AS-GLU groups were identified based on criteria of |log₂(fold change)| > 1 and p < 0.05. Second, a protein-protein interaction (PPI) network was constructed using the DEPs. Third, hub proteins were ranked using two centrality algorithms, Degree and Maximal Clique Centrality (MCC), implemented in the CytoHubba plugin. Fourth, the intersection of the top 20 proteins from both algorithms yielded 16 candidate hub genes. Finally, among these candidates, OPN, RAPGEF1, and MYL7 were selected for further validation based on their documented roles in vascular inflammation, integrin signaling, and smooth muscle function, processes centrally involved in atherosclerosis pathogenesis. (PNG) [file pone.0354719.s003.png]

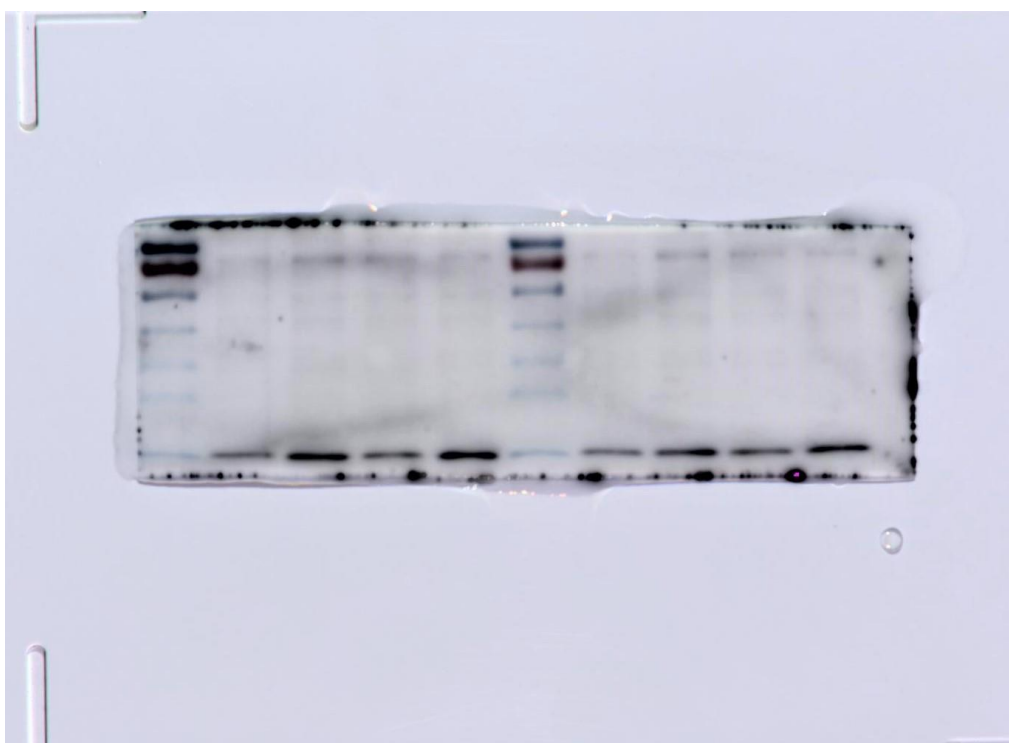

MYL7(18KD)-1,2

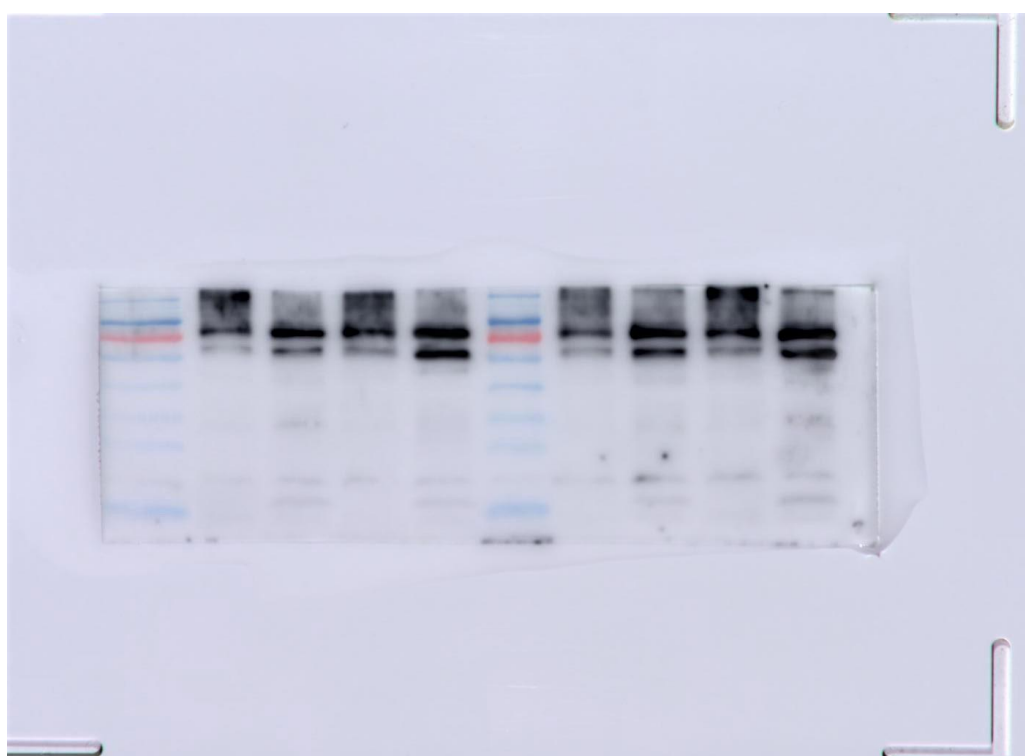

OPN(66KD)-3,4

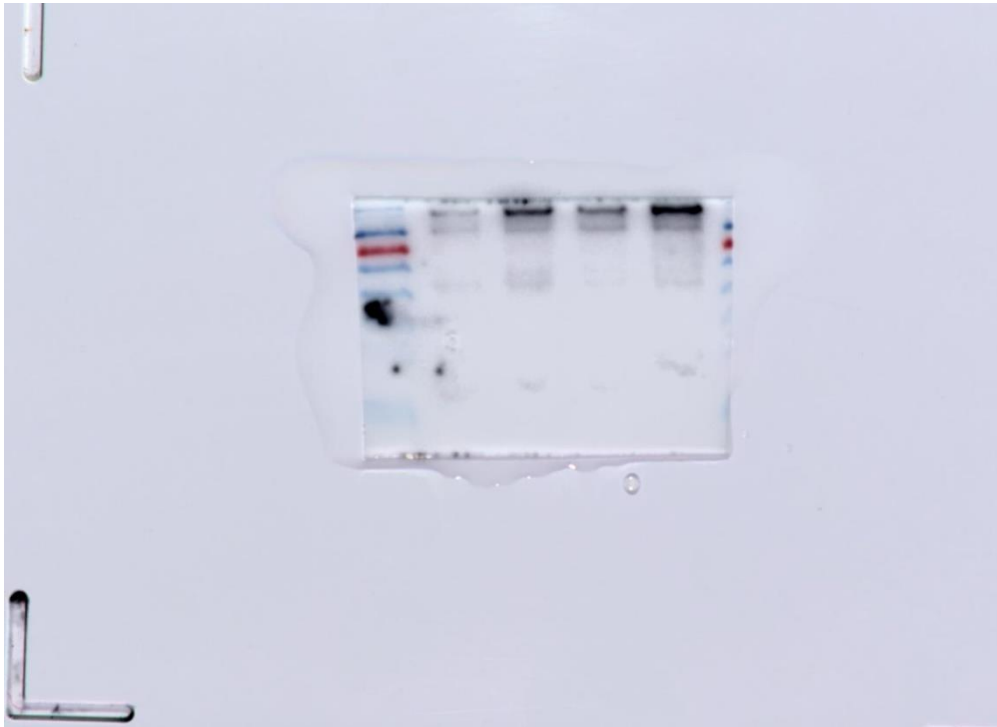

RAPGEF1(140KD)-5

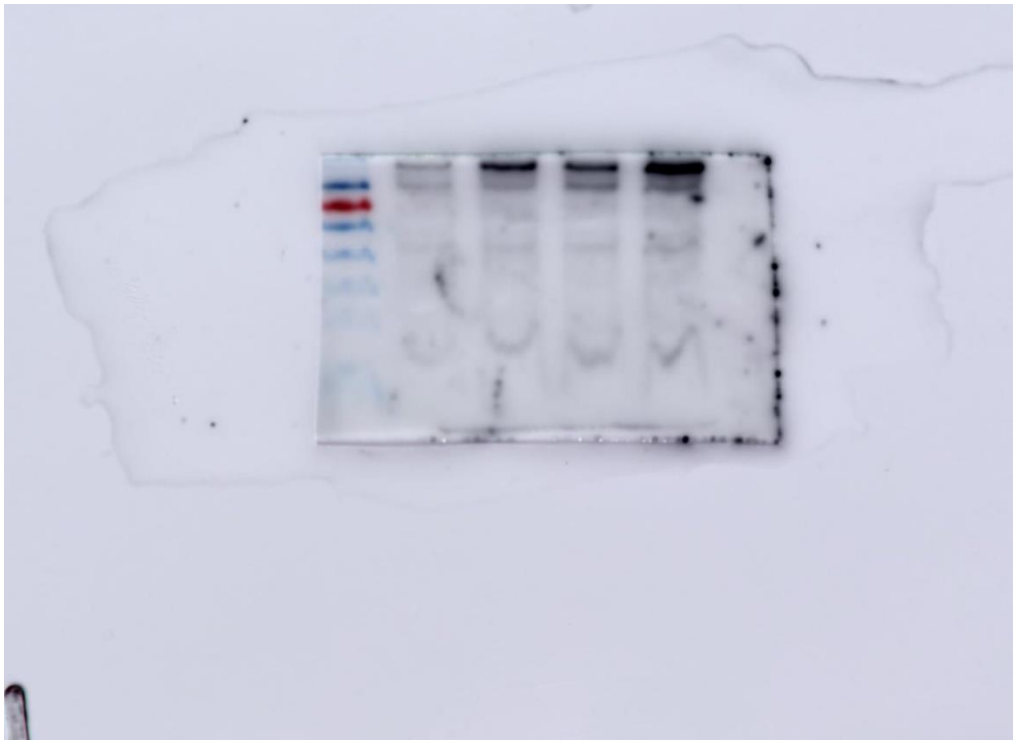

RAPGEF1(140KD)-6

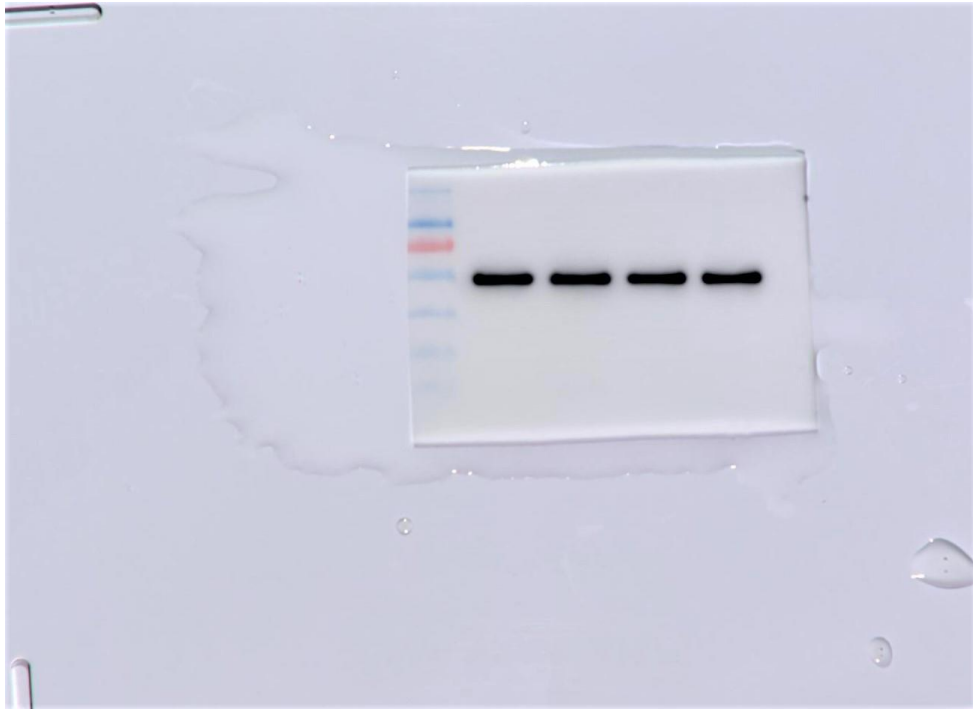

$\beta$  -tubulin(55KD)-1

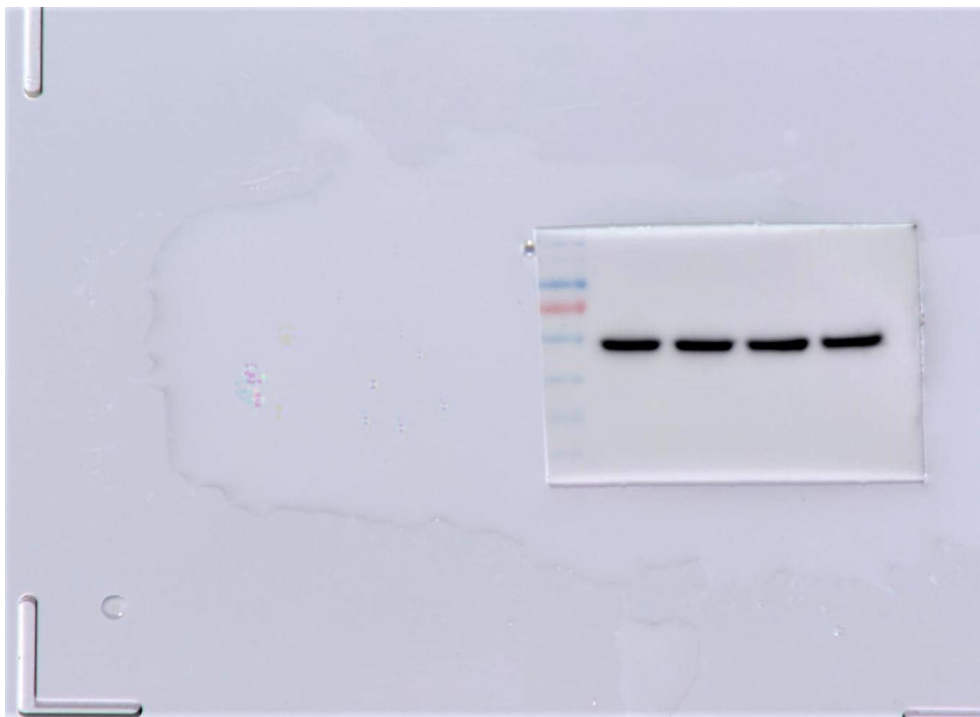

$\beta$  -tubulin(55KD)-2



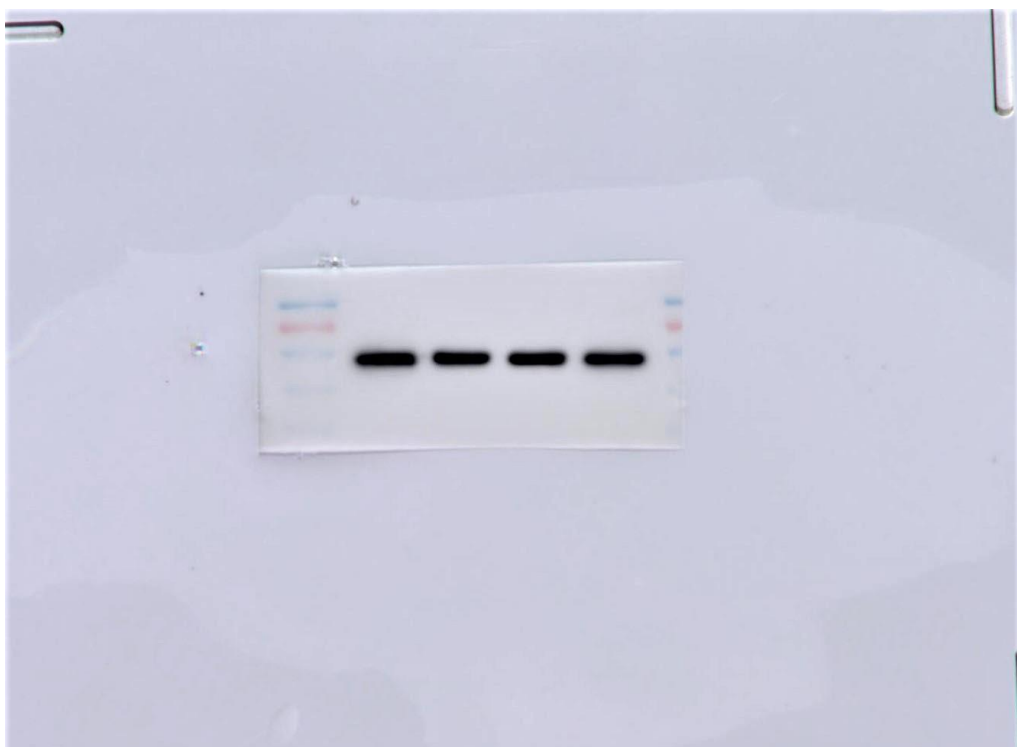

$\beta$  -tubulin(55KD)-5

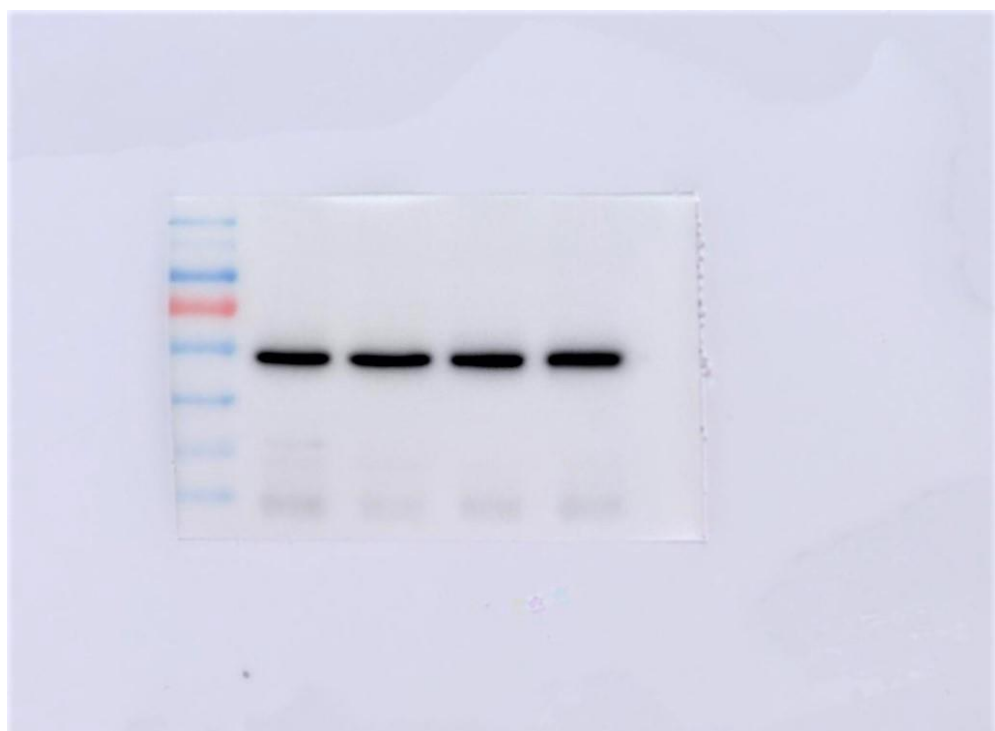

$\beta$  -tubulin(55KD)-6

Supplement: S1 File — (PDF) [file pone.0354719.s007.pdf]
